# Circulating cell-free DNA levels measured by a novel simple fluorescent assay are predictive for outcome of severe sepsis

**DOI:** 10.1186/cc10640

**Published:** 2012-03-20

**Authors:** A Douvdevani, A Avriel, M Paryente Wiessman, V Novack, Y Almog

**Affiliations:** 1 Soroka University Medical Center and Ben-Gurion University of the Negev, Beer-Sheva, Israel

## Introduction

Circulating cell-free DNA (CFD) was found to be a predictor of outcome in severe sepsis and septic shock [1]. The standard CFD assays are work-intensive and not practical for routine clinical laboratory use. We have recently developed a new simple, fast and reliable assay for CFD measurement. The aim was to evaluate the association between admission levels of CFD and severe sepsis outcome in patients hospitalized in intensive care utilizing the new assay.

## Methods

Seventy-six patients diagnosed with severe sepsis hospitalized in the ICU were enrolled in the study. Serum CFD levels were measured upon admission and after 72 hours using the SYBR-Gold rapid direct fluorescent assay [2]. Primary outcome was 28-day mortality. Logistic regression analysis of CFD quintiles adjusted for baseline comorbidities and severity of the disease was utilized.

## Results

Out of those diagnosed with severe sepsis, 28 (36.8%) have died either during hospitalization or within 28 days of admission to the ICU. Decedents had higher APACHE II score on admission (median 24.5 vs. 17.5, *P *= 0.140). Similarly their admission CFD levels were higher than in survivors (median 3,712 vs. 1,974, *P *= 0.001). Spearman's correlation analysis showed significant correlation between APACHE II score and CFD level on admission (ρ = 0.315, *P *= 0.007). ROC curve for APACHE II score and CFD level on admission for prediction of 28-day mortality showed area under the curve of 0.59, 95% CI 0.44 to 0.74 (*P *= 0.208), for APACHE II score; and area under the curve of 0.73, 95% CI 0.60 to 0.86 (*P *= 0.001), for CFD level on admission. The study group was divided into quintiles by CFD levels of admission. The 28-day mortality rate was 12.5% in the CFD lowest quintile and 60.9% in the highest quintile. Logistic regression analysis showed that adjusted for age, sex and APACHE II score CFD divided into quintiles was significantly associated with death at 28 days, OR = 1.83 per quintile (95% CI 1.12 to 2.98, *P *= 0.015).

## Conclusion

By using a simple fluorometric assay, we were able to measure CFD levels in severe septic patients. CFD levels were found to be an independent predictors for 28-day mortality. We believe that CFD is an objective, reliable and integrative prognostic marker that will allow fast evaluation of intensive care patients and predicting mortality.
